# A randomised clinical trial of subgrouping and targeted treatment for low back pain compared with best current care. The STarT Back Trial Study Protocol

**DOI:** 10.1186/1471-2474-9-58

**Published:** 2008-04-22

**Authors:** Elaine M Hay, Kate M Dunn, Jonathan C Hill, Martyn Lewis, Elizabeth E Mason, Kika Konstantinou, Gail Sowden, Simon Somerville, Kanchan Vohora, David Whitehurst, Chris J Main

**Affiliations:** 1 Arthritis Research Campaign National Primary Care Centre, Primary Care Sciences, Keele University, Staffordshire, ST5 5BG, UK

## Abstract

**Background:**

Back pain is a major health problem and many sufferers develop persistent symptoms. Detecting relevant subgroups of patients with non-specific low back pain has been highlighted as a priority area for research, as this could enable better secondary prevention through the targeting of prognostic indicators for persistent, disabling symptoms. We plan to conduct a randomised controlled trial to establish whether subgrouping using a novel tool, combined with targeted treatment, is better than best current care at reducing long-term disability from low back pain.

**Methods/Design:**

We will recruit 800 participants aged 18 years and over with non-specific low back pain from 8–10 GP practices within two Primary Care Trusts in Staffordshire, England. Our primary outcome measures are low back pain disability and catastrophising. Secondary outcomes include back pain intensity, global change, leg pain, fear avoidance, anxiety, depression, illness perceptions, patient satisfaction, overall health status and cost-effectiveness. Data will be collected before randomisation, and 4 and 12 months later. Participants are randomised to receive either newly developed interventions, delivered by trained physiotherapists and targeted according to subgroups defined by tool scores, or best current care.

**Discussion:**

This paper presents detail on the rationale, design, methods and operational aspects of the trial.

**Trial registration:**

Current Controlled Trials ISRCTN37113406.

## Background

Back pain is a major health problem in the UK. Each year, approximately 3.5 million people develop back pain [1], and 7–9% of adults consult their GP.[2] Although most sufferers stop consulting their GP within 3 months, 60–80% still have pain or disability a year later.[3,4] Persistent back pain affects peoples' quality of life, their family and social relationships, and impairs their ability to work.[5] Consequently, back pain has a huge economic impact. [6-8]

### Evidence for Treating Low Back Pain in Primary Care

Primary care guidelines for managing low back pain (LBP) [9-11] have been developed within a biopsychosocial framework which recognises that pain is influenced by both tissue pathology and psychological factors, as well as the social context in which the pain occurs. An estimated 85% of LBP consulters will have non-specific LBP, for which diagnostic labelling is discouraged, and treatment depends on the health care providers' preferences and clinical experience.[12] The fundamental question of "who will do best with which treatment" remains unanswered despite a number of high quality primary care-based randomised trials of treatment options for LBP in the last 2 years. [13-18]

### Subgrouping and Targeted Treatment for Low Back Pain

Findings from primary care studies contrast with specialist settings, where there is randomised controlled trial evidence that cognitive behavioural approaches help selected groups of patients with chronic back pain. [19,20] The problem, therefore might be that treatments are not reaching appropriate patient groups, and their effect is diluted by the inclusion of heterogeneous patient populations. Researchers are now questioning the appropriateness of considering non-specific back pain patients to be a homogenous group [21,22], and ways of identifying subgroups of patients who might benefit from specific therapies has been recommended.[23] There is growing evidence that psychosocial factors are particularly useful for predicting individuals who will develop chronic back pain, but in primary care these are difficult to spot and often go unrecognised.[17,24] Even when primary care clinicians do recognise psychosocial influences, they may be unable to manage them effectively (without additional training).[25] Researchers in some settings have developed clinical prediction rules to identify patients who are more likely to respond to specific treatments.[26,27] Other groups have developed and validated screening tools that identify patients at risk of work absence.[21,28] Early indications show these approaches to be promising, with improved outcomes when subgrouping is used to guide treatment decision-making.[29] Although it is recognised that these types of studies may improve the management of back pain patients, the implementation of such systems remains unclear, and research on the identification and treatment of subgroups of patients is highlighted as the "main challenge" for the treatment of LBP.[23]

### Development of the Subgrouping Tool

In response to this challenge, a simple-to-use LBP subgrouping tool for use in primary care – the STarT Back Tool – has been developed. [30-32] This tool classifies patients into 3 categories for targeted treatment, based on the presence of potentially modifiable physical and psychological prognostic indicators for persistent, disabling symptoms, identified through 9 questions. Patients are classified as "low risk" of future disabling LBP if they score positively on fewer than 4 questions. The remainder are then subdivided into "medium risk" (physical and psychosocial indicators for poor outcome, but without high levels of psychological indicators) and "high risk" (high levels of psychological prognostic indicators with or without physical indicators).

### Development of the Targeted Treatment Interventions

Alongside the development of the STarT Back tool, we have developed targeted treatments for patients allocated to the low, medium and high-risk subgroups; these have been designed to address the specific modifiable prognostic indicators identified by the tool. Evidence-based assessment and treatment approaches for LBP patients have been agreed and follow a "stepped-care" format. The focus of the interventions is directed towards the secondary prevention of disabling back pain. A three-month pilot study completed in September 2006 demonstrated that the study methods were feasible and acceptable in a LBP primary care population consulting their GP and seeking treatment. They also showed that it is feasible to recruit and treat the numbers of patients required for the trial within the set timeframe.

### Trial Objectives

The primary objective of this Trial is to compare the overall effectiveness of a "subgrouping for targeted treatment" approach with "best current care" (non-targeted) physiotherapy practice, over a 12 month period, for LBP. The targeted treatments are delivered by specially trained community physiotherapists.

Secondary objectives are: a) to investigate the change in prognostic indicators separately for each subgroup (high, medium and low risk) in the "targeted treatment" arm compared with the change in the corresponding subgroup in controls receiving "best current care" (non-targeted) physiotherapy practice; and b) To evaluate the cost effectiveness of the new model of care compared with "best current care".

## Methods

### Setting

Participants will be recruited from 8–10 general practices within the Keele GP Research Partnership. This Partnership includes a network of 30 practices that are committed to high quality consultation recording using Read Codes.[33] The population in this locality is primarily urban with some rural and inner city areas, and is therefore broadly representative of the population in the UK. Community Back Pain Clinics, through which patients can be recruited to the Trial, are held at two Primary Care Centres within this locality.

### Ethical approval

Ethical approval was obtained from the North Staffordshire Local Research Ethics Committee in February 2007 (ref number 07/Q2604/5).

### Eligibility criteria

Male and female subjects aged 18 years and above who seek care for LBP with or without associated leg pain are eligible to take part. Participants must speak and understand English and be willing and able to give full, informed written consent. The exclusion criteria are: potentially serious pathology (e.g. cauda equina compression, inflammatory arthritis, malignancy etc), serious co-morbidity, psychiatric illness or personality disorder; spinal surgery in the last 6 months; pregnancy; already receiving treatment other than GP care for this episode of back pain; and inability to attend regular physiotherapy appointments.

### Recruitment

Participants are identified when they consult their GP, practice nurse or the local Physiotherapy Direct Access service (Physio Direct) for LBP. At the GP practices, previously validated back pain Read Codes will be used to identify patients.[2] When a clinician enters one of these Read Codes, a pop up screen will remind them that the patient will be invited to a Community Back Pain Clinic. At this point the clinician can choose to "opt out" if the patient is considered to be inappropriate for referral to the Clinic, and/or is ineligible to take part in the Trial. The remaining patients are given an information sheet about the Clinic. One participating GP practice uses the local "Physio Direct" service, which offers direct access for patients with back pain, without the need for an initial consultation with their GP. Patients from this GP Practice who telephone the Physio Direct service instead of consulting their GP are initially assessed over the phone by a physiotherapist. Potentially eligible patients will be told about the Community Back Pain Clinic by the assessing physiotherapist and sent a clinic information sheet. All patients identified from the GP practices and Physio Direct are mailed a letter inviting them to telephone for an appointment at the Community Back Pain Clinic, along with a patient information leaflet about the Trial and a baseline questionnaire. Patients who telephone are given an appointment at one of the Community Back Pain Clinics. At the clinic, patients see a research nurse who explains the Trial in detail. For those patients interested in taking part, the nurse then goes through the written informed consent process. The research nurse is blinded to the patient's treatment allocation throughout the trial. All patients are assessed by an experienced physiotherapist at the clinic, regardless of whether they choose to take part in the Trial or not.

### Baseline assessment

All patients invited to the Community Back Pain Clinic are requested to complete the postal questionnaire and those who consent to take part in the study complete a further self report questionnaire in clinic. The questionnaires include the STarT Back Tool [32], age, gender, educational attainment and the outcome measures listed in Table 2 at time point zero.

### Treatment allocation

**Figure 1:**
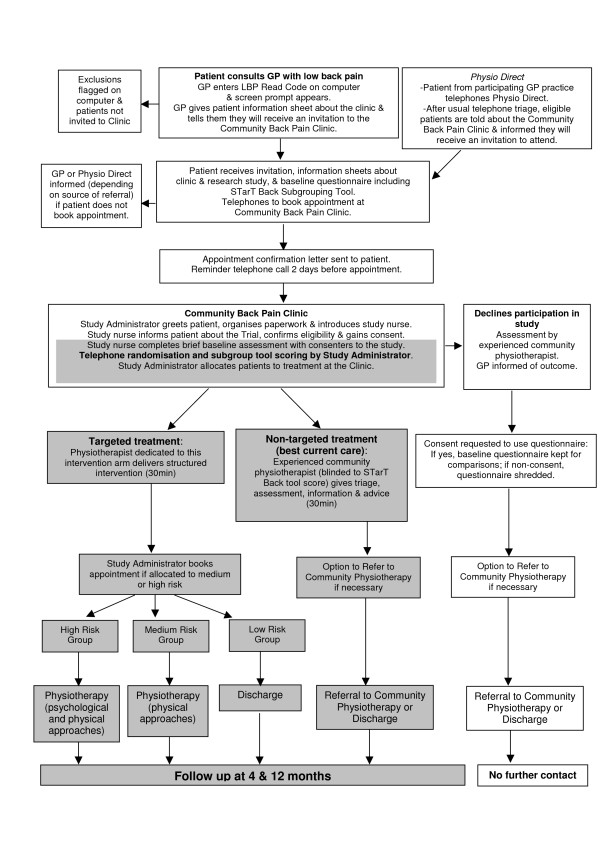
STarT Back Trial Flow Chart.

Following completion of the baseline questionnaire, patients who consent to take part in the Trial are randomly allocated to one of the two treatment arms: "targeted treatment" or "best current care" (see Figure 1). After calculating the patient's STarT Back tool score from the postal questionnaire, the clinic administrator telephones the Primary Care Musculoskeletal Research Centre randomisation service.[15,34] Patients are randomised using a stratified block randomisation method according to Centre and risk subgroup. Random allocation is on a 2:1 (targeted treatment: best current care) ratio basis with a block size of three. This gives three possible permutations of treatment allocation per block: AAB; ABA; BAA. The research nurse in clinic is blinded to a patient's treatment allocation.

### Interventions

#### 1) Targeted Treatment

All patients randomised to 'targeted treatment' receive an initial 30-minute protocolised intervention with a specially trained study physiotherapist. Patients receive reassurance about the benign nature of their pain, and simple messages of advice around issues such as pain relief, appropriate activity levels and the role of further investigations. The physiotherapist is guided by the results of the patient's STarT Back tool score so that specific concerns can be identified and addressed on an individual basis. To reinforce key messages the 'Back Book' [35] is given, together with an information sheet of local contacts for exercise venues such as swimming pools, gyms and exercise on prescription, and self-help groups. A 15-minute educational video based on The Back Book, called 'Get Back Active' [36] is shown to patients to reinforce key messages. Patients allocated to the 'low risk' group receive no further treatment in addition to this 30 minute clinic appointment.

For patients allocated to receive the medium and high risk subgroup interventions, the study administrator arranges further appointments with specially trained physiotherapists within two weeks of their clinic date. Treatments for the medium and high risk groups were developed after reviewing available literature and guidelines to identify best practice treatment, and are delivered during a target time of six weeks. The focus was to provide secondary prevention of disabling back pain through the provision of treatment targeted at the specific modifiable prognostic indicators identified by the STarT Back tool (see below).

Patients in the 'medium risk' group present with predominantly physical prognostic indicators (disabling back pain, referred leg pain and co-morbid pain), without high levels of psychosocial distress. The 'medium risk' group package of care therefore, targets physical characteristics using a range of physiotherapy techniques delivered over 6, 30-minute sessions. Management includes an assessment and differential diagnosis (particularly for patients with referred leg pain), and treatment designed to reduce disability related to future episodes of low back pain.

The 'high risk' group physiotherapy treatment is tailored for patients who present with high psychosocial indicators such as anxiety and fear. Therapists are given extra training to deliver this package, which helps them identify and address pain related psychological distress through the use of appropriate cognitive behavioural strategies. Goal setting, where realistic treatment goals are negotiated with patients using a Goal Attainment Scale.[37] The components of the targeted treatments are outlined in Table 1.

**Table 1:** Components of Targeted Treatment

**Low, medium and high risk groups **(30-minute structured intervention)
• Evidence-based assessment of LBP presentations, according to current guidelines.
• Advice on back care emphasising positive messages about activity, pain relief and work.
• Patients are given a copy of "The Back Book" [35] and see a 15-minute video based on The Back Book entitled "Get Back Active" [36].

**Medium and high risk groups**
• Treatment according to STarT Back assessment and treatment algorithms and STarT Back physiotherapy manual.
• Evidence-based physiotherapy techniques addressing 'signs and symptoms' in non-specific LBP presentations, according to current recommendations from guidelines and high quality clinical trials.
• Evidence-based advice, education and reassurance for symptoms and management, according to guidelines and current literature within the biopsychosocial model.

**High risk group**
• Assessing and addressing biopsychosocial risk factors by adopting cognitive behavioural principles to address unhelpful beliefs and behaviours.

**Table 2:** Outcome Measures

	**Domain**	**Measures**	**Time Point (months)**
Primary	Pain and disability	Roland and Morris Disability Questionnaire [38]	0, 4, 12
	Catastrophising	Pain Catastrophising Scale [39]	0, 4, 12
Secondary	Back Pain	Pain intensity: 0–10 numerical rating scales of least & average pain in last 2 wks & current pain	0, 4, 12
		Duration – time since month back pain free [42]	0
	Back-related Leg symptoms	-Leg pain in last week	0, 4, 12
		-Location of leg pain	0, 4, 12
		-Description of pain	0, 4, 12
		-Presence of Numbness/parasthesia	0, 4, 12
	Pain elsewhere	Self complete manikin for bodily pain in last 4 weeks [43]	0, 12
	Global change	Compared to symptoms at baseline	4, 12
	Kinesiophobia	Tampa Scale Kinesiophobia [44]	0, 4, 12
	Illness perceptions	Musculoskeletal Illness Perceptions Questionnaire (MUSC-IPQ(R))^†^	0, 4, 12
	Anxiety/depression	Hospital Anxiety and Depression Scale [45]	0, 4, 12
	Health-related quality of life	Short Form 12 (version 2) [46]	0, 4, 12
	Patient satisfaction	-With current symptoms	0, 4
		-With information received	4
		-With care received	4
		-With back pain knowledge	4
		-Would have same treatment again	4
		-Rating of overall results of care	4
	Patient expectations	-Expectations for LBP status	0
		-Extent to which expectations were met	4
	Employment details	-Current employment status	0, 12
		-Satisfaction with employment	0, 12
		-Current/most recent job title	0, 12
		-Work loss due back problem	0, 12
		-Work loss due to other health problems	0, 4, 12
		-Work performance	0, 12
	Health care	-Primary care consultations	4, 12
	resource use	-Secondary care attendances (NHS and private)	4, 12
		-Additional health care practitioners (NHS and private)	12
		-Prescription and over-the-counter medicines and treatments	12
	Preference-based measure of health-related quality of life	EQ-5D [47]	0, 4, 12

^† ^Instrument in development, Personal Communication, Nadine Foster.

#### 2) Best Current Care

Patients randomised to the control arm have a 30-minute consultation with an experienced community physiotherapist (not involved in the 'targeted treatment' arm) who is blinded to the results of the STarT Back tool. A management plan is formulated according to normal clinical practice. This may include referral to community physiotherapy if thought necessary.

Patients from both "targeted treatment" and "best current care" arms are advised that they can access their GP for ongoing care in the usual way, and that they should contact their GP if their condition worsens.

### Audit of interventions

At the clinic, both study physiotherapists and community physiotherapists complete case report forms for all patients participating in the trial. This records assessment findings relating to leg pain and documents medication and advice given in clinic. Physiotherapists who deliver the targeted treatment use a standard proforma to record the number of treatment sessions each participant receives, plus details about the treatments delivered at each session. They also receive regular clinical mentoring and supervision sessions with expert members of the STarT Back Trial team who are responsible for training.

### Outcome measures

As the main focus of this trial is secondary prevention of chronic back pain, primary clinical outcomes are changes in the main physical and psychosocial risk factors for chronicity: disability (Roland-Morris Disability Questionnaire (RMDQ)) [38] and pain catastrophising (Pain Catastrophising Scale) [39] (see Table 2). The RMDQ is a recommended disability measure [40] that it is widely used in low back pain studies in Primary Care, and pain catastrophising has been shown to be an important predictor of quality of life.[41]

Secondary outcome measures are described in Table 2. These were selected to fulfil recommendations on outcome data [40], and to capture more fully some aspects of back-related leg pain, psychological measures and quality of life. [42-47] The health economic evaluation will consist of a cost-utility analysis, with utility measured by the EuroQol EQ-5D.[47] To estimate the costs of the study interventions and consider the resource implications for the targeted treatment approach, the 12-month self-report questionnaire will collect resource use data for back pain-specific and generic health care (direct costs) and details of patients work absence due to low back pain (indirect costs). All resources will be costed using UK national averages from freely available sources. [48-51]

### Sample size

The primary outcome measure for this trial is the Roland and Morris Disability Questionnaire (RMDQ) at 12-months. A difference of 2.5 points in RMDQ change scores is considered to be a minimum clinically important difference in the RMDQ.[52] Our sample size calculation is based on two separate hypothesis tests: (i) to test superiority of targeted treatment over best current care for high and medium risk subgroups; (ii) to test non-inferiority of the targeted treatment compared to best current care in the low risk subgroup. For the first hypothesis, a subgroup sample size of 160 patients (107 and 53 per arm based on a 2:1 allocation ratio) would enable detection of a between-group difference of 2.5 RMDQ points given 80–90% power, 5% (two-tailed) significance level, and a conservative standard deviation of 5 points. For the second hypothesis, the targeted treatment will be concluded as being non-inferior to best current care if the lower boundary of the 95% confidence interval for the difference in mean scores for targeted treatment minus best current care (among low-risk participants) is above the threshold of -2.5 points (equivalent to a one-sided test with significance level of 2.5%); 160 patients (107 and 53 per arm) allocated on a 2:1 basis would be able to demonstrate non-inferiority with 80–90% power. Pilot study data estimated that 25%, 50% and 25% of participants would be in the high, medium and low risk subgroups, respectively. These figures were used to extrapolate the total sample size requirement of 640 patients (160 in the high and low risk subgroups and 320 in the middle risk subgroup), which would ensure that we would have at least 80% power for carrying out all three subgroup analyses. 800 participants would need to be recruited to allow for a conservative 20% loss to follow up. Based on a 2:1 random allocation basis, we would therefore expect about 533 and 267 to be recruited to the "targeted treatment" and "best current care" arms, respectively.

### Data analysis

The primary analysis will be by intention to treat; a per protocol analysis will be performed as a sensitivity analysis. Estimates of treatment effects with 95% confidence intervals ("targeted treatment" minus "best current care") will be calculated. Statistical tests (analysis of covariance (ANCOVA) for numerical outcomes; logistic regression for categorical outcomes) will be performed adjusted for imbalances in the baseline scores. Analyses will be carried out at each time point (4 and 12 months) for primary and secondary outcome measures; the primary endpoint is 12-month follow up. Analyses will be carried out to compare outcomes in patients randomised to "targeted treatment" or "best current care" separately for each of the three subgroups (high, medium and low risk) of patients classified according to the subgrouping tool.

An incremental approach will be used in the economic evaluation. The primary aim is to estimate and compare the societal costs associated with the study interventions and relate this to the difference in the number of quality-adjusted life years (QALYs). QALYs will be calculated by applying area-under-the-curve techniques to the EQ-5D scores at baseline, 4 months and 12 months.[53] Differences in costs and QALYs will be expressed using the incremental cost-per-QALY ratio, with uncertainty addressed using bootstrapping techniques, cost-effectiveness planes and acceptability curves.[54,55] The robustness of the results will be tested by conducting a number of sensitivity analyses; namely, the consideration of alternative perspectives (e.g. adopting an NHS perspective), the effect of incorporating 'generic' health care resource use and the application of multiple imputation techniques to deal with missing data.

An independent data monitoring committee will monitor the trial every 6 months. There will be no interim analyses.

## Conclusion

The STarT Back Trial will investigate the clinical and cost effectiveness of a novel subgrouping and targeted treatment approach for patients with non-specific low back pain in Primary Care. Using the results of the Pilot study, we estimate that we can recruit 800 patients to the trial in 12–18 months if we involve 8–10 GP practices from the Keele GP Research Partnership and Physio Direct and run 3 Community Back Pain Clinics a week. Trial recruitment commenced in July 2007 and is currently on target to close before January 2009. Follow up is targeted for completion by February 2010 and results should be finalised for publication in summer 2010.

The primary objective of the trial is to compare the clinical outcomes of subgrouping and targeted treatment with best current care over a 12 month period. The secondary objectives are to compare the clinical outcomes for each of the three subgroups compared with best current care over 12 months; and to evaluate the cost effectiveness of subgrouping and targeted treatment compared with best current care at 12 months.

## Competing interests

The authors declare that they have no competing interests.

## Authors' contributions

All authors participated in the design of the Trial and the drafting of the manuscript. All authors have read and approved the final manuscript.

## Pre-publication history

The pre-publication history for this paper can be accessed here:


